# Food commodity pipeline management in transitional settings: challenges and lessons learned from the first USAID food development program in South Sudan

**DOI:** 10.9745/GHSP-D-13-00018

**Published:** 2013-08-14

**Authors:** Hannah Tappis, Shannon Doocy, Stephen Amoako

**Affiliations:** a Johns Hopkins Bloomberg School of Public Health, Department of International Health, Baltimore, MD, USA; b Adventist Development and Relief Agency (ADRA) International, Silver Spring, MD, USA

## Abstract

Despite decades of support for international food assistance programs by the U.S. Agency for International Development (USAID) Office of Food for Peace, relatively little is known about the commodity pipeline and management issues these programs face in post-conflict and politically volatile settings. Based on an audit of the program's commodity tracking system and interviews with 13 key program staff, this case study documents the experiences of organizations implementing the first USAID-funded non-emergency (development) food assistance program approved for Sudan and South Sudan. Key challenges and lessons learned in this experience about food commodity procurement, transport, and management may help improve the design and implementation of future development food assistance programs in a variety of complex, food-insecure settings around the world. Specifically, expanding shipping routes in complex political situations may facilitate reliable and timely commodity delivery. In addition, greater flexibility to procure commodities locally, rather than shipping U.S.-procured commodities, may avoid unnecessary shipping delays and reduce costs.

## Abstract (precis)

Efficient and reliable commodity transport is critical to effective food assistance in development settings as well as in emergency situations. Increasing the flexibility of U.S. government Title II food assistance program procurement regulations and more comprehensive contingency planning could improve the effectiveness of these programs in non-emergency settings with high food insecurity and political volatility.

## BACKGROUND

Food assistance programs, which are complex in any setting, frequently face unanticipated logistical challenges in delivering the correct types and quantities of food in humanitarian situations, as well as in non-emergency situations where food insecurity can nevertheless be severe. Despite decades of support for such programs by the U.S. Agency for International Development (USAID) Office of Food for Peace (FFP), relatively little is documented about commodity pipeline and management issues in post-conflict settings beyond the internal program data used for monitoring and reporting. Moreover, new factors have come into play, including the increasingly prolonged nature of conflict and the global food, finance, and fuel crises. This evolution of the global and local environments in which food assistance programs function has implications for foreign assistance policy and practice. This case study aims to contribute to the evidence base by documenting the challenges faced and lessons learned from a food assistance program in South Sudan, an area undergoing a transition from relief to development programming at a time of substantial political change and instability.

Changed global and local contexts require a review of food assistance policy and practice, particularly commodity pipeline issues in transitional settings.

Under the Food for Peace Act of 1954,* the USAID Office of Food for Peace is tasked with managing programs under Title II of the Trade portion of the Farm Bill, which provides for donation of U.S. agricultural commodities and humanitarian assistance to meet emergency and non-emergency food needs in other countries. Food aid provided under Title II is primarily targeted to vulnerable populations in foreign countries in response to malnutrition, famine, natural disaster, civil strife, and other extraordinary relief requirements. Title II resources can also be used to provide non-emergency (development) assistance to address chronic malnutrition, boost agricultural productivity and incomes, and help build resilience in the most food-insecure countries with high levels of stunting and poverty.^1^ In terms of program design, Title II emergency and development program activities are similar; the key difference is that emergency food programs often provide rations that are designed to meet a significant proportion, if not all, of a household's nutritional needs, whereas development programs incorporate a range of other activities and provide fewer targeted rations.^2^

USAID has provided food aid to Sudan (including the southern area now recognized as the Republic of South Sudan) for more than 20 years. Until 2010 this assistance was solely in the form of emergency aid.^3^-^4^ The South Sudan Health, Nutrition and Empowerment (SSHiNE) program was the first Title II development food assistance program approved for implementation in Sudan and South Sudan. A consortium of nongovernmental organizations (NGOs) implemented the program. Its members consisted of Concern Worldwide, Food for the Hungry, Malaria Consortium, and the Adventist Development and Relief Agency (ADRA). ADRA, the lead agency in the consortium, has more than 50 years of emergency and development food assistance experience, including more than 20 years of experience in South Sudan and a history of successful non-emergency food assistance program implementation in post-conflict Bosnia, Mozambique, Nicaragua, and Zimbabwe.

**Figure 1.**
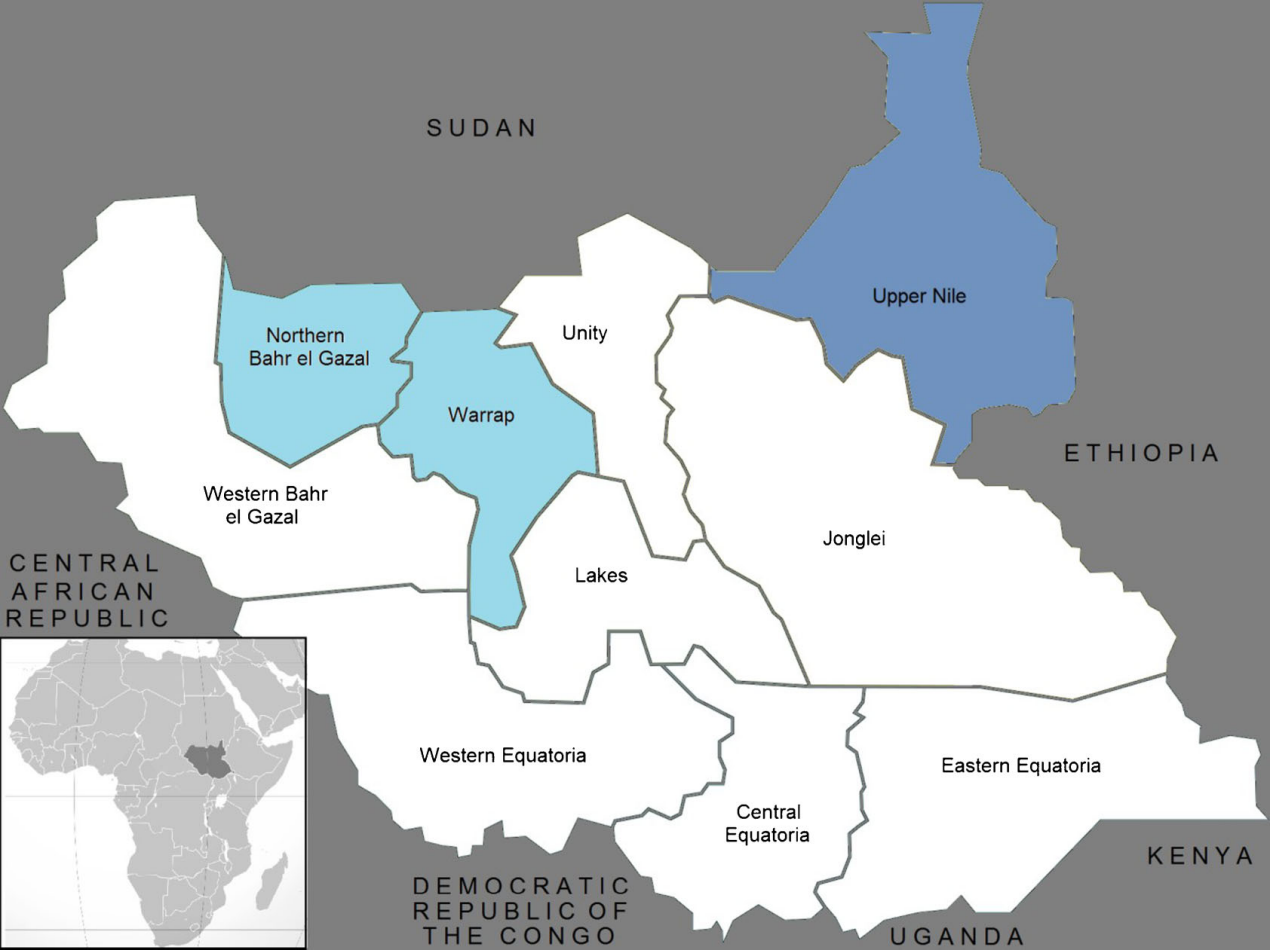
Map of SSHiNE Program Areas The project was initially planned for Northern Bahr el Gazal, Warrap, and Upper Nile States but was implemented only in Northern Bahr el Gazal and Warrap because of cost, logistics, timing, and security considerations.

The program was initially approved for implementation from July 2010 through June 2013 in the three South Sudanese states of Northern Bahr el Ghazal, Warrap, and Upper Nile (Figure 1). The launch of the project marked not only a transition in U.S. strategies for food assistance, linking relief to development in the region, but also coincided with a period of unprecedented political, economic, and social transition in the country. These transitions included the referendum for self-determination that took place in January 2011, the subsequent expiration of the Comprehensive Peace Agreement (CPA), and transition to independence in July 2011.

## METHODS

As proposed, the SSHiNE program was a multiyear assistance program intended to reach 500,752 direct and 504,250 indirect beneficiaries, with a direct impact on the nutrition status of 40,420 children at an estimated total cost of US$55 million. The scope of the program was later reduced to cover a smaller geographic area, eliminating programming in Upper Nile State. This reduced the total number of beneficiaries to 251,904 direct and 250,000 indirect beneficiaries, with a direct impact on the nutrition status of 20,150 children, and shortened the implementation period by 1 year, ending in June 2012.

This case study is the result of regular review of ADRA's commodity tracking system throughout the project period and in-depth interviews conducted with 13 key program staff and stakeholders in South Sudan and Washington, D.C. between January and May 2012. Participants included SSHiNE senior management staff (all expatriate), and South Sudanese commodity and logistics coordinators, food warehouse managers, and food distribution supervisors employed by ADRA, Concern Worldwide, and Food for the Hungry, as well as program staff from ADRA headquarters who were involved in logistics, procurement, commodity distribution, and overall program management. Additional participants included representatives from the World Food Programme and UN Logistics Cluster in South Sudan and from USAID FFP in both South Sudan and Washington, D.C.

Interviews were conducted by a member of the Johns Hopkins Bloomberg School of Public Health (JHSPH) research team funded by a sub-award to conduct operational research within SSHiNE program implementation. Informed consent was obtained orally prior to initiating all interviews. The study protocol was reviewed by the Institutional Review Board at JHSPH, which determined that this was not research on human subjects.

## FINDINGS

**Figure 2.**
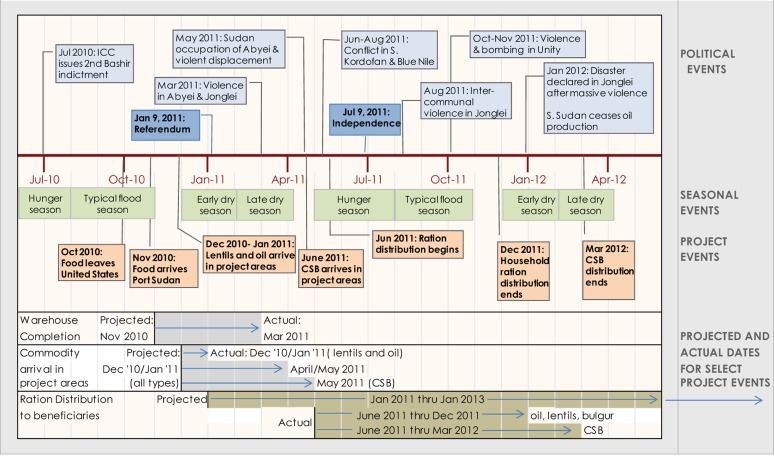
Timeline of Significant Events, July 2010–June 2012 Abbreviations: CSB, corn-soya blend.

During the course of project implementation, relations between northern and southern Sudan worsened dramatically, causing many delays and unforeseen obstacles in commodity procurement, transport, storage, management, and distribution, as illustrated in Figure 2. SSHiNE program staff and stakeholders identified 3 major challenges to program implementation: (1) procuring the required food baskets; (2) transporting the food from port to project areas; and (3) ensuring proper storage facilities.

### Challenge #1: Procurement of Complete Food Basket

Since 2010, USAID has encouraged non-emergency, multiyear food assistance program implementers to adopt a preventive approach to reducing malnutrition in children under 2 years of age. This approach has been shown to be more effective in reducing the prevalence of stunting, underweight, and wasting in the context of Title II-funded programs than recuperative nutrition interventions alone.^5^-^6^

Core components of the Preventing Malnutrition in Children Under 2 Approach (PM2A), which was a central component of the SSHiNE program, include rations targeted to pregnant and nursing women and children under 2 years of age, which are conditional on participation in behavior change interventions; behavior change communication; and preventive and curative health and nutrition services for women and children delivered according to national protocols. In addition, the PM2A package often includes a household ration to supplement the household food supply, to prevent sharing of targeted rations, and/or to provide an incentive for participation in preventive program activities, such as clinic visits and growth monitoring. PM2A is implemented in food-insecure areas with high prevalence of stunting or underweight; it targets pregnant and breastfeeding women and children 6–23 months of age because they are the subgroups that are most vulnerable nutritionally.^7^

#### The Issue of Genetically Modified Corn-Soya Blend

For the SSHiNE project, the PM2A ration package proposed by ADRA and approved by USAID FFP consisted of bulgur, lentils, vegetable oil, and corn-soya blend (CSB). Title II development food assistance funding requires that all donated commodities be of U.S. origin. The bulgur, lentils, and vegetable oil components of the ration were provided in-kind by the U.S. government, with the consignment for the first year of the project loaded on U.S. flagged vessels in Texas, destined for Port Sudan, in late October 2010. The CSB approved for donation by the U.S. government was a genetically modified (GM) variety and thus restricted from import into Sudan, a scenario that is not unprecedented in Sudan and is not surprising given the increasing number of nations banning the import of GM foods.^8^

Assumptions about how South Sudan would regulate genetically modified food led to critical delays in the procurement of a key food ration for vulnerable women and children.

At the time the SSHiNE project was awarded, the USAID mission in Sudan was to allocate a portion of the project funding as direct assistance through the USAID office in Juba to cover procurement, transport, warehousing, and distribution costs during the first year of the project for non-GM CSB. USAID representatives and authorities in South Sudan thought that in following years an independent South Sudan would loosen its regulations on GM importation and that subsequent consignments of CSB could be provided by the U.S. government in kind. However, in order for South Sudan to join the Common Market for South and Eastern Africa (COMESA), a stricter regulatory framework for GM foods was required, in alignment with regulations throughout most of East Africa.^9^-^10^

It was a challenge to identify a single supplier that could provide the needed quantity of non-GM CSB in the time frame required for the first year of programming. At the start of the project, ADRA identified potential suppliers of non-GM CSB in Italy and Belgium (both of which were regular suppliers of CSB to the World Food Programme). Before any contracts were awarded, the USAID mission in Juba suggested that ADRA consider procuring regionally in East Africa rather than from Europe. After multiple rounds of advertising for suppliers, a Kenyan supplier was eventually identified. However, the commodity order was not placed until March 2011, which was 3 months after food distribution was to begin (see proposed and actual project milestones in Figure 2). Having prior arrangements with regional mills in Kenya or Tanzania might have reduced the delays in CSB procurement.

### Challenge #2: Transport of Food Commodities from Port to Project Areas

#### Port Clearance

During many years of war, southern Sudan could viably receive goods only from its southern neighbors Uganda and Kenya. Commodities would arrive via Kenya's port city of Mombasa, with subsequent land transfer through Kenya, often by way of Uganda, to South Sudan. However, following the signing of the CPA in 2005, Port Sudan, located in northern Sudan on the Red Sea, also became a viable entry point for food commodities destined for the south, albeit not without challenges.

The USAID-commissioned 2009 Bellmon Estimation for Title II (BEST) report found that importation of food commodities through Port Sudan would be more cost-effective than through Mombasa.^11^ Therefore, Port Sudan was identified as the primary seaport to receive food commodities for the SSHiNE project. This proved to be an inauspicious decision: The entire shipment of 1,650 metric tons of bulgur was held at Port Sudan for more than 3 months. During this time the Sudan Port Health Authority, Customs Authority, and Meteorology Organization conducted more than 30 tests to ascertain the appropriateness of the bulgur for human consumption before they granted clearance.

The use of a northern port to receive commodities despite political tensions meant a 3-month delay in transit to the south.

The ADRA Khartoum office, which was responsible for clearing the commodities in Sudan, engaged with USAID and local officials in efforts to get the bulgur released. Despite persistent efforts by ADRA and USAID offices in both Khartoum and Juba to negotiate release of the commodities, authorities claimed that such extensive testing was necessary because bulgur was a new import crop for the Republic of Sudan. However, history suggests that this is not the case. Between 2005 and 2009, Sudan was among the top 5 importers of bulgur worldwide.^12^ Project stakeholders speculate that the customs clearance was prolonged with the sole intention of obstructing aid destined for South Sudan. Lack of clarity on import/export procedures from Sudan to South Sudan is a documented source of concern during the SSHiNE program period that forced some humanitarian organizations to use alternative supply corridors.^13^ In the case of SSHiNE, the transit of bulgur at port took over 100 days, compared with about 10 days under normal conditions.

#### Inland Transport (Port Sudan to Project Areas)

**Figure 3.**
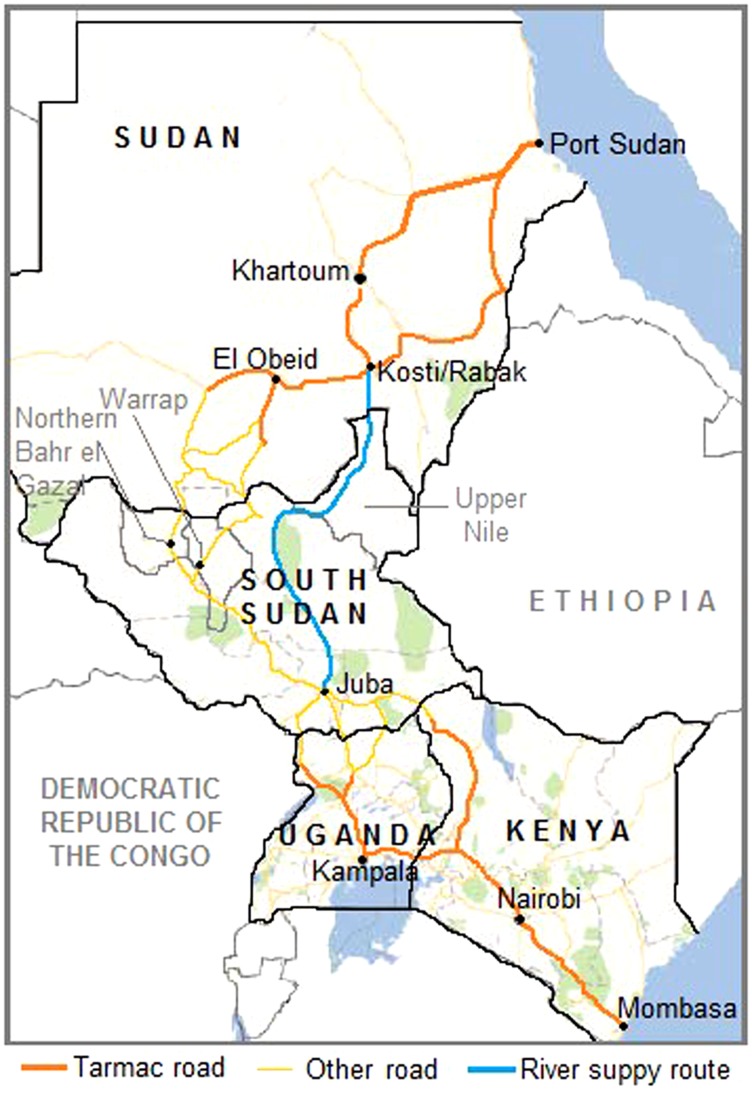
Key Transport Routes From Port Sudan to SSHiNE Program Areas and Alternative Routes Through Mombasa

All commodities were transferred from Port Sudan to trucks for inland transport immediately upon customs clearance. For the lentils and vegetable oil, this occurred in late November 2010, with 26 trucks setting off on the approximately 2-week journey of more than 2,300 km from Port Sudan via El Obeid to Northern Bahr el Ghazal and Warrap states. An additional 16 trucks set off on the 1-week trip (1,200 km) to Kosti, where commodities would be loaded on river barges for a 2-week trip to Upper Nile (Figure 3).

When the trucks carrying the lentils and vegetable oil arrived in project areas in December 2010, the bulgur consignment was still being held by customs agents at Port Sudan. By the time the bulgur was finally cleared through customs at Port Sudan in mid-March 2011, northern troops had begun amassing around Abyei, and the security situation along the route had deteriorated substantially. Commodity transport delays and budget shortfalls resulting from rising implementation costs led ADRA to request that project activities be conducted in only Warrap and Northern Bahr el Ghazal, 2 contiguous states, thereby cancelling implementation in the more geographically challenging Upper Nile State, which is accessible only during the dry season. The 26 trucks with bulgur destined for Warrap and Northern Bahr el Ghazal were dispatched to their end destinations in South Sudan. Since the request to cancel implementation in Upper Nile had not yet been approved, the 16 additional trucks were dispatched to Kosti, the Nile river port, pending USAID authorization to redirect the commodities to the other 2 project states.

Commodity transport delays and rising costs due to deteriorating security led to a decision to limit the focus of project activities to 2 states.

Although the vast majority of the commodities arrived intact, the overland journey from Port Sudan to Warrap and Northern Bahr el Gazal was not without obstacles. The truck convoys encountered many official and unofficial roadblocks, where soldiers or local authorities demanded bribes in the form of money or “small gifts” of commodities from the trucks. The roads from El Obeid to Northern Bahr el Gazal, Warrap, and Kosti are technically considered “all-weather” roads; in practice, however, there are often substantial delays due to road conditions, in particular during periods of heavy rain. There are no truck weight restrictions or stoppages of movement during or following heavy rain; therefore, many trucks become stuck, bridges are damaged, and roads are impassible during the wet season despite major road rehabilitation and demining activities.^14^

Nearly all trucks arrived at their destinations intact. One truck was missing 87 bags (4.2 metric tons) of bulgur upon arrival. Another truck, carrying 1,100 bags (55 metric tons) of bulgur, drove over a landmine after crossing into Warrap state (between Mayom and Abeinhom towns), destroying all commodities on board, severely damaging the vehicle, and injuring the driver and his assistant. The Table summarizes transit losses along with other commodity tracking information.

**TABLE.** Commodities Procured, Stocked, and Distributed (metric tons)

	Commodity					
	Lentils	Vegetable Oil	Bulgur Wheat	Corn-Soya Blend	Total	Percentage of Total Commodity Purchased
Commodity purchased	219.00	159.45	1,641.35	340.00	2,359.80	–
Marine losses	–	0.11	11.85	–	11.96	0.5%
Transit losses	1.05	0.17	59.20	–	60.42	2.6%
Commodity stranded in Rabak	43.50	32.84	330.00	–	406.34	17.2%
Commodity received in project warehouses	174.90	126.33	1,240.30	340.00	1,881.53	79.7%
Commodity distributed	174.85	126.30	1,240.30	337.63	1,879.08	79.6%

Source: SSHiNE commodity tracking system reports.

#### Unanticipated Border Closures

By the time ADRA had received formal USAID approval to forego programming in Upper Nile and redirect commodities to Warrap and Northern Bahr el Ghazal, the border between north and soon-to-be independent South Sudan had been closed due to conflict over disputed territory in the Abyei region and worsening relations over unresolved CPA issues, such as border demarcation, fees for oil export via Sudan, and citizenship rights.^15^ The 405.89 metric tons of food commodities (330 metric tons of bulgur, 43.05 metric tons of lentils, and 32.84 metric tons of vegetable oil) initially destined for Upper Nile project areas remained stuck in Rabak for months while attempts were made to obtain permission from Sudanese authorities to allow food to be moved across the border. Finally, in April/May 2012, after over a year of negotiations with authorities in both Sudan and South Sudan, SSHiNE project management and USAID concluded that it would not be possible to secure the release of these stocks and instead distributed the commodities to Sudanese returnees (northern and southern) at an ADRA-operated way station in Kosti.

### Challenge #3: Proper Storage Facilities

Section 403 of the 2008 Food for Peace Act mandates that adequate storage facilities be available in the host country when commodities arrive to prevent spoilage or waste.^16^ Given the lack of adequate warehousing facilities in any of the project areas, ADRA ordered 20 portable warehouses from W. Giertsen HallSystem in Norway at the beginning of the project. ADRA did not have experience in South Sudan with installation of portable warehouses, and forecasting their delivery and installation was a challenge. The original timeline for importation, delivery, and construction of the warehouses prior to the arrival of commodity shipments in December 2011 was unrealistic. The materials for warehouses in Warrap and Northern Bahr el Ghazal did not arrive at Port Mombasa, Kenya until January 2011, after the consignments of lentils and vegetable oil had already arrived in the project areas. Until the warehouse materials arrived in the project areas and warehouse construction was completed in March 2011, ADRA was forced to store the approximately 222 metric tons of lentils and vegetable oil in school buildings, containers, and guarded open spaces near project offices. This resulted in unanticipated expenses in temporary storage and quality control.

## DISCUSSION

Many of the challenges described above, such as unwarranted customs delays at Port Sudan and border closures between northern and southern Sudan before independence (and between Sudan and South Sudan after), might have been anticipated, given the area's long history of logistical challenges faced by food aid programs pre-independence. Indeed, other studies have recognized that:

South Sudan is an operational context fraught with challenges for aid agencies including lack of suitable partner organizations, high staff turnover, diversion, severe logistical constraints caused by rains and flooding in the rainy season and the near impossible task of targeting assistance.^17^

The SSHiNE program encountered additional challenges stemming from the fact that it was the first U.S.-funded development food assistance initiative in the country and that it was implemented during a unique period of instability and political transition to independence. Still, the nature of the challenges highlights the need for additional contextual analysis, contingency planning, and innovative approaches in settings undergoing political change or a transition from relief to development programming. Several aspects of Title II programming could benefit from a critical assessment:

### “Emergency” Versus “Non-emergency” Designation

Re-examination of the criteria for designation of “emergency” versus “non-emergency” food assistance programs is warranted. In the face of the political uncertainty surrounding the referendum for self-determination, the end of the CPA and the transition to independence, the long-term security situation in South Sudan was at best unpredictable at the time that the request for proposals for the food assistance program was released. The situation was further complicated by the lack of rain in many parts of the country in 2011, including the SSHiNE implementation area, which diminished the harvest season severely and worsened food insecurity. By the beginning of 2012, the situation had so deteriorated that it was designated a crisis (Integrated Phase Classification 3), with the potential for further deterioration to a humanitarian emergency.^13^ The difficulty of making accurate long-term security predictions in many transition settings requires innovative strategies, such as the adaptation of voucher, cash transfer, and insurance schemes increasingly used in emergency settings^18^-^19^ and greater donor flexibility for advance financing or use of prepositioned commodities.^20^

Strategies used in emergencies, including donor flexibility on financing, should be adapted to transition settings where security cannot be predicted accurately.

### A Cost-Benefit Approach to Timely Commodity Delivery

Expanded alternatives for procurement, shipping, and transport, such as those proposed as part of U.S. government food aid reforms for fiscal year 2014, could facilitate reliable and timely commodity delivery—a central tenant of effective food assistance programming. Importation through secondary and tertiary routes, presumably involving different seaports and ports of entry into the destination country, would increase the reliability of commodity pipelines in a situation of political unrest, security concerns, and/or poor transportation infrastructure. This undoubtedly would come with additional costs. However, the SSHiNE experience suggests that the current approach of using the least costly port and inland-shipping routes may be inadequate if the situation threatens commodity losses or delays that could significantly alter the scope and impact of the program. A nuanced cost-benefit approach that allows for a more complete comparison of both the reliability and the constraints of different shipping routes, and the costs associated with each, may provide for more complete and timely delivery of commodities in complex political situations.

More options for procurement, shipping, and transport would make commodity pipelines more reliable in the face of security concerns or poor transportation infrastructure.

### Flexible Local Procurement

Greater flexibility should be considered for local and regional purchase of food commodities in settings with political instability or notable logistical challenges. Humanitarian and development practitioners have suggested that increased allowances for local and regional procurement may improve U.S. food assistance programming in several ways. Arrival delays of several months are typical when commodities are shipped from the United States, and ocean transportation on U.S.-flagged carriers is relatively costly. In contrast, food obtained via local procurement has been shown to reduce costs and improve timeliness of delivery, without disrupting local markets.^21^-^22^

Local procurement of fortified blend foods (FBF) is a common challenge, which in the case of the SSHiNE program resulted in delayed food distribution. Possible approaches to address this challenge at the system level include the development of planning models to better predict demand for FBFs, longer-term contracts for FBF vendors, and shipping of micronutrient premix to the region for local milling and fortification. Improving the capacity for fortification and processing in developing countries in the vicinity of emergency operations would coincide with USAID development goals as outlined in the Feed the Future Initiative.^2^

Local procurement is unlikely to become central program strategy since Title II food programming provides for the donation of U.S. agricultural commodities to meet humanitarian food needs in other countries.^23^ However, local purchase could be funded through strategic public-private partnerships, and allowances could be made for this alternative in certain contexts. Critics of local purchase maintain that it would undermine the coalition of commodity groups, private voluntary organizations, and shippers that support U.S. Title II programs and ultimately result in the reduction of U.S. food assistance.^24^ However, proposed food aid reforms would replace $250 million of Title II funding within development assistance to a Community Development and Resilience Fund, providing more flexibility to purchase foods locally and regionally while maintaining a significant portion of U.S. commodity purchases. Such reforms could be an effective means of minimizing the programmatic impacts of pipeline failure for commodities of U.S. origin while having limited implications for domestic interests in the United States.^25^

Local food purchase can minimize pipeline failure for commodities of U.S. origin with limited impact on U.S. domestic interests and without disrupting local markets.

### Contingency Planning to Account for Changing Policy

In post-conflict settings, potential changes in government policies, such as policy on imports of GM foods, require contingency planning. Given that young children are among the most nutritionally vulnerable and that preventive strategies are now the approach of choice for Title II development programming, the availability of commodities optimized for this age group is crucial if U.S. food aid programs are to contribute to improvements in child growth.

Many fortified blended foods, such as the different varieties of CSB or wheat-soya blend (WSB) have GM components, and this creates challenges for their widespread use in food aid programs, especially U.S. food aid, where commodities generally must be of U.S. origin. Many countries have expressed reservations about or banned the import of GM foods (including commodities imported for food aid programs).^26^ Planning for additional production of U.S.-origin non-GM CSB, local purchase of alternative fortified blended foods that may be more acceptable to beneficiary populations, or the use of more costly Ready-to-Use Supplementary Foods (RUSFs), which may be easier to target to intended beneficiaries, are approaches that could be used to address similar challenges in future food assistance programs.

In the SSHiNE program, critical challenges arose from uncertainty about the regulations that would be put in place post-independence and the pace at which political decisions would be made and protocols established. Inclusion of contingency plans at the proposal stage and joint development of alternative implementation strategies by implementing partners and FFP will be important to the success of U.S. food assistance programs operating in transitional contexts.

Contingency plans and alternative implementation strategies should be developed at the program proposal stage.

### Limitations

This study focuses on lessons learned from the South Sudan experience (based on the opinions of the interviewees) that have implications for food aid in similar situations. Including an analysis of organizational capacity and decision-making of the implementing organizations would have yielded a more comprehensive assessment of this specific situation, but such findings would not be generalizable. In addition, distinguishing between external, contextual factors and internal, organizational factors is sometimes challenging. For example, identifying and retaining qualified field staff is a major issue for NGO programs in South Sudan and an external factor that clearly influenced organizational capacity and decision making. At the same time, internal factors, such as the compensation package offered by the implementing organization may have exacerbated recruitment difficulties in an already challenging environment.

## CONCLUSION

Title II food assistance programs aim to meet humanitarian food needs and promote food security among vulnerable populations in foreign countries. The nature of the settings in which many of these programs operate is such that the challenges faced in commodity transport are frequent, extreme, and unpredictable. More flexibility in the procurement, transport, and management of commodities could increase the effectiveness of Title II food assistance programs. The proposed food aid reforms for fiscal year 2014, which will enhance the ability of NGOs to adapt implementation strategies to the local context, are an important step toward more responsive programming.

The U.S. government and international organizations continue to be committed to providing emergency and development assistance in conflict and disaster-affected contexts. Critical to the success of both short-term humanitarian response efforts and longer-term development initiatives is the ability of NGOs to anticipate challenges and implement contingency plans with rapid approval by donors. This flexibility, along with real-time adaptation strategies to address contextual challenges, local procurement of commodities, and attention to the need for acceptable and affordable supplementary foods, will contribute to improved delivery of U.S. food assistance abroad.

## Footnotes

* Commonly known as Public Law 480; reauthorized as the Federal Agriculture Improvement and Reform (FAIR) Act in 1996, which is commonly known as the 1996 Farm Bill.
